# Pattern of Recurrence After Platinum-Containing Definitive Therapy and Efficacy of Salvage Treatment for Recurrence in Patients with Squamous Cell Carcinoma of the Head and Neck

**DOI:** 10.3389/fonc.2022.876193

**Published:** 2022-07-04

**Authors:** Tetsuro Wakasugi, Thi Nga Nguyen, Shoko Takeuchi, Jun-ichi Ohkubo, Hideaki Suzuki

**Affiliations:** Department of Otorhinolaryngology—Head and Neck Surgery, University of Occupational and Environmental Health Japan, Fukuoka, Japan

**Keywords:** platinum-refractory recurrence, platinum-sensitive recurrence, platinum chemotherapy, recurrent or metastatic head and neck squamous cell cancer, NLR

## Abstract

**Background:**

In first-line systemic therapy for unresectable recurrent and/or metastatic squamous cell carcinoma of the head and neck (R/M SCCHN), regimens are generally selected by time-to-relapse with 6 months cutoff after platinum (Pt)-containing definitive therapy, Pt-refractory or Pt-sensitive recurrence, but clinical characteristics between Pt-refractory and Pt-sensitive recurrence of R/M SCCHN has not been fully investigated. This study aimed to evaluate pattern of recurrence and efficacy for salvage treatment for recurrence after Pt-containing definitive therapy for R/M SCCHN in a real-world setting.

**Methods:**

We retrospectively reviewed 150 patients treated with Pt-containing definitive therapy and analyzed the pattern of recurrence and efficacy of salvage therapy for 63 patients with R/M SCCHN.

**Results:**

Pt-refractory recurrence, Pt-sensitive recurrence, second primary cancer (SPC), and no relapse occurred in 23.3%, 18.7%, 14.7%, and 43.3% of patients, respectively. In the cases with distant metastatic recurrence, symptomatic recurrence was significantly more common in the Pt-refractory recurrence, while asymptomatic recurrence was significantly more common in the Pt-sensitive recurrence. The timing of detection of SPC was after 2 years in 59.0% of cases after the completion of definitive therapy and 63.6% of SPC were asymptomatic. There was a significant difference in ΔNLR2 (NLR after definitive therapy minus NLR at detection recurrence; *p* = 0.028) and in prognosis after the detection of recurrence for the overall population (*p* = 0.021), and for salvage treatment group (*p* = 0.023), and systemic therapy group (*p* = 0.003) between Pt-refractory and Pt-sensitive groups.

**Conclusions and Significance:**

Our analysis revealed the recurrence pattern after Pt-containing definitive therapy and showed the validity of dividing patients into Pt-refractory and Pt-sensitive recurrence with different prognosis in salvage therapy, especially systemic therapy.

## Introduction

Patients with local advanced squamous cell carcinoma of the head and neck (L/A SCCHN) are treated with definitive therapy by surgery or radiotherapy (RT). Additionally, platinum (Pt) agents are key drugs in systemic therapy and are used in induction chemotherapy (ICT), chemoradiotherapy (CRT), or postoperative CRT (POCRT). However, even with these multimodality treatments, more than 50% of patients receiving definitive treatment develop locoregional and/or distant relapse within the first 2 years of initial treatment, and have a poor prognosis (1).

The treatment strategy for recurrent or metastatic (R/M) SCCHN is dictated by the availability of locoregional treatment or sensitivity to Pt agents defined by the time-to-relapse in case of unresectable disease. Currently, two programed cell death-1 (PD-1) inhibitors, pembrolizumab (Pembro) and nivolumab (NIVO), were used as first-line systemic therapy worldwide for patients who are not amenable to local treatment. Pembro is used as a first-line chemotherapy for the Pt-sensitive recurrence or untreated patients without feasibility of local treatment (2), while nivolumab is one of the most widely used regimens in the Pt-refractory patients with unresectable R/M SCCHN (3). In other words, the choice of Pembro and NIVO is determined only by the time-to-relapse, platinum-refractory, or platinum-sensitive recurrence, not based on the biological characteristics of recurrent cancer. Furthermore, to the best of our knowledge, the frequency of Pt-refractory and Pt-sensitive recurrence has not been reported, and also the pattern of recurrence and clinical course limited to only after Pt-containing definitive therapy has not been sufficiently examined.

Here, we retrospectively evaluated the pattern of recurrence after Pt-containing definitive therapy and investigated the efficacy of salvage treatment for recurrence in patients with R/M SCCHN.

## Materials and Methods

### Study Population

We retrospectively reviewed the medical records of patients treated with definitive therapy between January 2010 and December 2019 at the University of Occupational and Environmental Health, Japan. In the present study, the inclusion criteria were as follows: (a) primary tumor site located in the head and neck; (b) histologically proven SCC; (c) stage III or IV according to the American Joint Committee on Cancer Staging System (Seventh edition); (d) treated with Pt-containing definitive therapy by CRT or surgery followed by POCRT; (e) diagnosis of R/M SCCHN. The exclusion criteria were as follows: (a) primary tumor site located in nasopharynx; (b) p16 positive oropharyngeal cancer.

### Definitive Therapy and Follow-Up Visit

Pt-containing definitive therapy was performed by surgery followed by POCRT or CRT. In cases, in which ICT with TPF (docetaxel/cisplatin/5-FU) was performed, Pt-containing definitive therapy was subsequently performed. The Pt agent in combination with RT was used daily carboplatin 25 mg/m^2^ until August 2015, and weekly cisplatin 40 mg/m^2^ or triweekly cisplatin 80–100 mg/m^2^ after September 2015. A Pt agent was administered to patients with normal renal function above Cockcroft-Gault 60. The definitive radiotherapy dose was 70–72 Gy and the adjuvant radiotherapy dose was 60–66 Gy. Nutritional support was provided using prophylactic percutaneous endoscopic gastrostomy (PEG) before definitive radiotherapy. In cases without prophylactic PEG, nasogastric tube was used when patients had inadequate oral intake. All patients were supported with nutritional supplements. After the Pt-containing definitive therapy, patients were followed with a medical examination every 1–2 months and imaging examination by computed tomography (CT) including the neck and chest every 3–4 months for the first 2 years and followed with imaging examination by CT every 6 months from the third to the fifth years.

### Patient Characteristics

We collected the patients’ characteristics at definitive therapy and the detection of recurrence including age, sex, Eastern cooperative Oncology Group (ECOG) performance status (PS), primary tumor site, T and N status, disease stage, definitive therapy for primary tumor, radiotherapy, platinum agent used in definitive therapy, induction chemotherapy, nutrition support, body weight (BW), and neutrophil-to-lymphocyte ratio (NLR), symptoms at the time of detection of recurrence, detection modalities, and extent of recurrent disease.

### BW and NLR

BW measurement and peripheral blood test were performed before definitive therapy, after definitive treatment, and at the time of detection of recurrence. NLR was calculated as the total neutrophil count divided by the total lymphocyte count. BW and NLR were defined as pre-BW and pre-NLR before definitive therapy, post-BW and post-NLR after definitive therapy, and rec-BW and rec-NLR at the detection recurrence, respectively. ΔBW1 and 2 were defined as pre-BW minus post-BW and post-BW minus rec-BW, respectively. ΔBW1% and 2% were calculated as a percentage of pre-BW and post-BW, respectively. ΔNLR1 and 2 were defined as pre-NLR minus post-NLR and post-NLR minus rec-NLR, respectively.

### Salvage Treatment

In the salvage treatment, surgery was the first choice if resectable. In the case of unresectable disease, RT was selected if the field was not previously irradiated, and systemic therapy was performed if local treatment was difficult. Systemic therapy includes conventional cytotoxic chemotherapy, molecular targeted agents (cetuximab), and immune checkpoint inhibitors (ICIs) such as nivolumab or pembrolizumab. Nivolumab and pembrolizumab have been used in patients since March 2017 and December 2019, respectively. Best supportive care (BSC) alone was performed when it was difficult to treat with these salvage therapies. Their median follow-up period was 470 days (range 19–3,516 days).

### Statistical Analysis

No statistical sample size calculations were conducted. Fischer’s exact test, the *χ* ^2^-test, independent two-sample *t*-tests, or one-way analysis of variance were used to evaluate differences between two or three independent groups. The overall survival (OS) rate was estimated and plotted using the Kaplan−Meier method and compared using the stratified log-rank test. The data were censored on December 31, 2021. OS was calculated from the date of the start of salvage therapy to the date of death or the last confirmed date of survival. Risk factors of recurrence and of survival for salvage therapy were evaluated by estimating hazard ratios (HRs) and 95% confidence interval (CI) using univariate and multivariate COX proportional hazards models. SPSS version 27 software (SPSS Inc., Chicago, IL, USA) was used for all statistical analyses. Statistical significance was defined as a *p* value <0.05.

## Results

**Figure 1:**
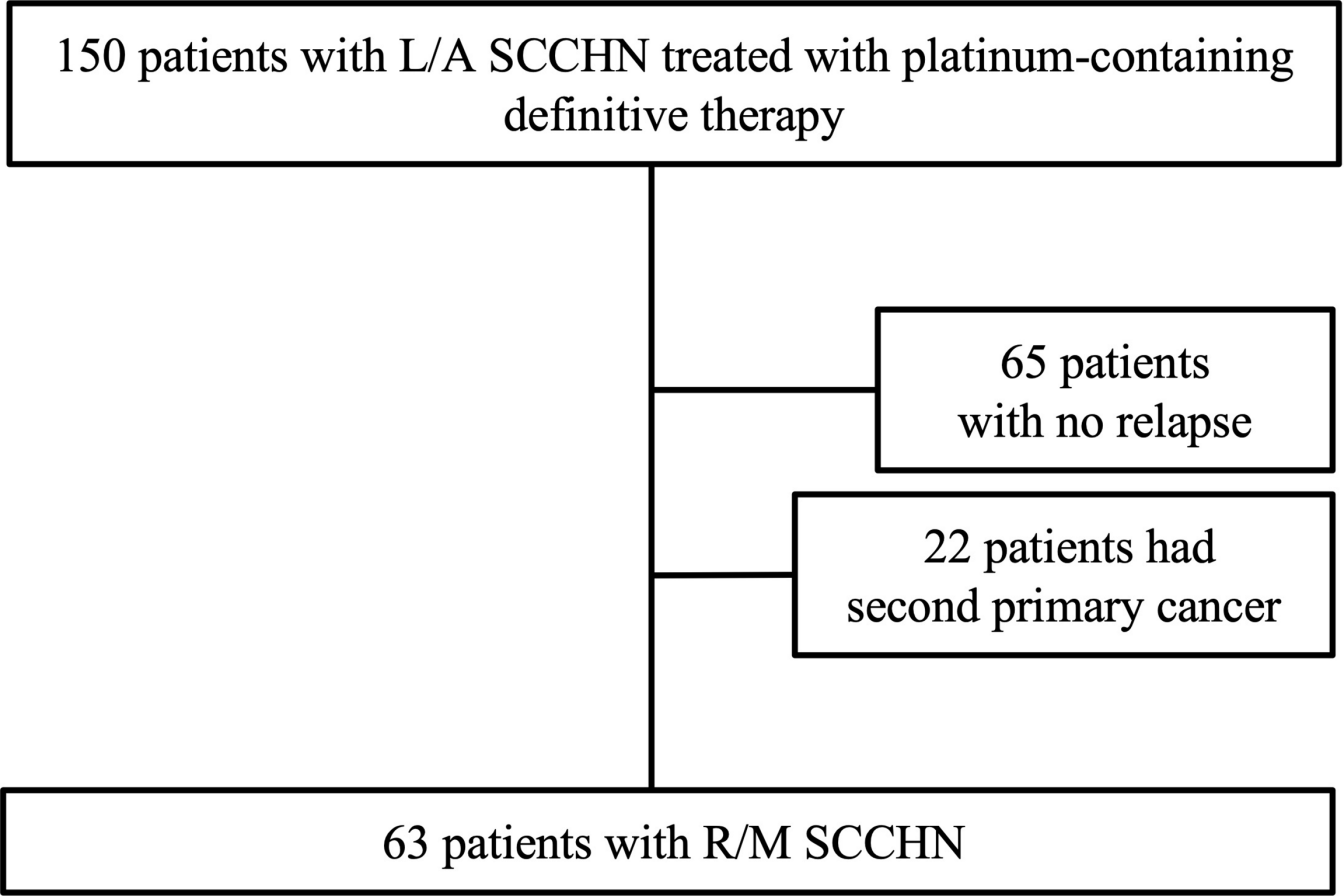
Diagram showing the process of patient selection. L/A SCCHN, local advanced squamous cell carcinoma of head and neck; R/M, recurrent and/or metastatic.

**Figure 2:**
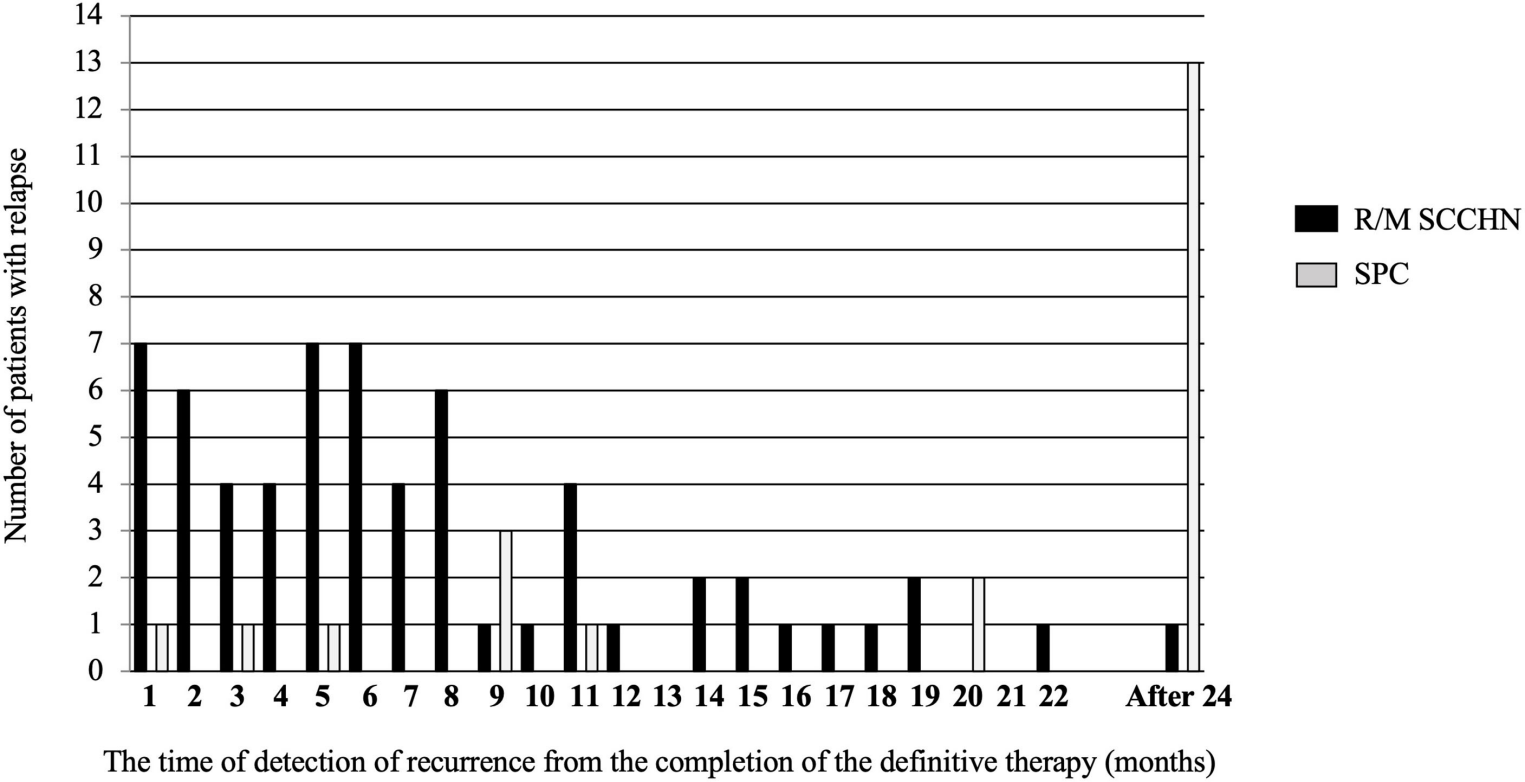
Histogram for the time of the detection of recurrence after definitive therapy.

Of the 150 patients with L/A-SCCHN treated with Pt-containing definitive therapy, 65 patients (43.3%) who were free of recurrence, and 22 patients (14.7%) with a second primary cancer (SPC), and 63 patients (37.2%) with locoregional and/or distant metastasis as shown in **Figure 1**. Histogram of the time of detection of recurrence from the completion of the Pt-containing definitive therapy was shown in **Figure 2**. Median time of detection of R/M SCCHN and SPC were 166 days (95% confidence interval [CI]: 117–215) and 894 days (95% CI: 330–1,457), respectively. Thirty-five patients (23.3%) of 150 patients who treated with Pt-containing definitive therapy had relapse within 6 months after Pt-containing definitive therapy (Pt-refractory recurrence) and 28 patients (18.7%) of 150 patients who treated with Pt-containing definitive therapy had relapse more than 6 months after Pt-containing definitive therapy (Pt-sensitive recurrence). Fifty-one patients (81.0%) and 61 patients (96.8%) had a relapse within first 1 year and 2 years, respectively. Thirteen (59.0%) of the 22 patients with SPC occurred after the second year. Primary lesion of the SPCs included esophagus in 9, lung in 6, stomach in 2, prostate in 2, and others in 3 patients. **Table 1** presents the patient characteristics at Pt-containing definitive therapy. There was no significant difference between no relapse, R/M SCCHN, and SPC groups except primary tumor site. In the no relapse group, primary tumor sites were less hypopharynx and more larynx compared to R/M SCCHN and SPC. In contrast, in the R/M SCCHN group, primary tumor sites were less oropharynx.

**Table 1:** Characteristics at platinum-containing definitive therapy.

	No relapse (n=65), n (%)	R/M SCCHN (n=63), n (%)	SPC (n=22), n (%)	P-value
**Median age, years (range)**	65 (29–81)	63 (48-78)	63 (47-75)	0.863
**Sex**				0.467
Male	50 (76.9)	53 (84.1)	19 (86.4)	
Female	15 (23.1)	10 (15.9)	3 (13.6)	
**Primary tumor site**				0.035*
Oral cavity	10 (15.4)	15 (23.8)	1 (4.5)	
Oropharynx	22 (33.8)	10 (15.9)	7 (31.8)	
Hypopharynx	15 (23.1)	26 (41.3)	11 (50.0)	
Larynx	11 (16.9)	4 (6.3)	1 (4.5)	
Nasal cavity and paranasal sinus	7 (10.8)	8 (12.7)	2 (9.1)	
**T status:**				0.072
T1	0	4 (6.3)	2 (9.1)	
T2	21 (32.3)	10 (15.9)	8 (36.4)	
T3	18 (27.7)	17 (27.0)	6 (27.3)	
T4	26 (40.0)	32 (50.8)	6 (27.3)	
**N status:**				0.744
N0	15 (23.1)	15 (23.8)	4 (18.2)	
N1	9 (13.8)	7 (11.1)	6 (27.3)	
N2	38 (58.5)	37 (58.7)	11 (50.0)	
N3	3 (4.6)	4 (6.3)	1 (4.5)	
**Disease stage：**				0.175
III	17 (26.2)	11 (17.5)	8 (36.4)	
IV	48 (73.8)	52 (82.5)	14 (63.6)	
**Definitive therapy for primary tumor：**				0.556
Surgery followed by POCRT	22 (33.8)	27 (42.9)	5 (22.7)	
CRT	43 (66.2)	36 (57.1)	17 (77.3)	
**Platinum agent used in definitive therapy:**				0.764
Cisplatin	32 (49.2)	33 (52.4)	10 (45.5)	
Carboplatin	33 (50.8)	30 (47.6)	12 (54.5)	
**Induction chemotherapy:**				0.985
Yes	22 (33.8)	21 (33.3)	7 (31.8)	
No	43 (66.2)	42 (66.7)	15 (68.2)	
**Prophylactic PEG:**				0.898
Yes	12 (19.0)	13 (20.6)	5 (22.7)	
No	53 (81.5)	50 (79.4)	17 (81.8)	

CRT, chemoradiotherapy; POCRT, postoperative chemoradiotherapy; R/M SCCHN, recurrent or metastatic squamous cell carcinoma of head and neck; SPC, second primary cancer; PEG, percutaneous endoscopic gastrostomy.

*, significant difference.

Patients’ characteristics at the detection of R/M SCCHN and SPC (**Table 2**), and BW and NLR (**Table 3** and **Supplementary Data 1**) were analyzed to evaluate potential clinical indicators of recurrence. As shown in **Table 2**, there was no significant difference with respect to PS, examination used for the detection of recurrence, extent of recurrent disease, and symptoms between R/M SCCHN and SPC groups. Recurrence was most frequently detected by imaging examination, although 23.8% and 36.4% of recurrences in R/M SCCHN and SPC groups were detected by medical examination, respectively. Asymptomatic recurrence was observed in 39.7% of patients in R/M SCCHN group and 63.6% of those in SPC group. In the cases with distant metastatic recurrence, symptomatic recurrence was significantly more common in the Pt-refractory recurrence, while asymptomatic recurrence was significantly more common in the Pt-sensitive recurrence in **Table 4**. Among patients with symptomatic recurrence, pain was the most common symptom in both cohorts. Time-to-relapse had significant difference between R/M SCCHN and SPC groups, and 86.4% of SPC occurring after 6 months. As shown in **Supplementary Data 1**, there was no significant difference between no relapse, R/M SCCHN, and SPC groups in pre-BW, post-BW, ΔBW1, ΔBW1(%), pre-NLR, post-NLR, and ΔNLR1. R/M SCCHN group was divided into the Pt-refractory and Pt-sensitive and analyzed adding BW and NLR at the detection of recurrence in **Table 4**. There was a significant difference in ΔNLR2 (*p* = 0.028) between the Pt-refractory and Pt-sensitive cohorts. BW decreased and NLR increased after the completion of definitive treatment. The results indicate the following: at the detection of recurrence, BW tended to decrease slightly in both groups, while NLR decreased in the Pt-sensitive recurrence and remained unchanged in the Pt-refractory recurrence. Clinical indicators of recurrence were analyzed using univariate analysis, but no significant factors were found in **Supplementary Data 2**.

**Table 2:** Characteristics of R/M SCCHN and SPC at the detection of recurrence.

	R/M SCCHN (n=63), n (%)	SPC (n=22), n (%)	P-value
**PS：**			0.376
PS 0	34 (54.0)	8 (36.4)	
PS 1	21 (33.3)	10 (45.5)	
PS 2	7 (11.1)	2 (9.1)	
PS 3	1 (1.6)	2 (9.1)	
**Time-to-relapse：**			0.001*
Before 6 months	35 (55.6)	3 (13.6)	
After 6 months	28 (44.4)	19 (86.4)	
**Examination used for detection of recurrence：**			0.254
Imaging examination	48 (76.2)	14 (63.6)	
Medical examination	15 (23.8)	8 (36.4)	
**Extent of recurrent disease：**			
Locoregional only	29 (46.0)	–	
Distant metastasis	34 (54.0)	–	
**Symptoms:**			0.052
Asymptomatic	25 (39.7)	14 (63.6)	
Symptomatic	38 (60.3)	8 (36.4)	
Pain	20	1	
Dysphagia	2	1	
Anorexia	5	0	
Cough	3	0	
Dyspnea	2	2	
Others	6	4	

R/M SCCHN, recurrent and/or metastatic squamous cell carcinoma; SPC, second primary cancer; PS, performance status; *, significant difference.

**Table 3:** BW and NLR before definitive therapy, after definitive therapy, and at the detection of recurrence.

	Pt-refractory Average ± SD	Pt-sensitive Average ± SD	P-value
Pre-BW (kg)	57.8 ± 10.7	57.6 ± 11.9	0.937
Post-BW (kg)	52.3 ± 9.5	53.0 ± 9.3	0.811
Rec-BW (kg)	50.5 ± 10.2	52.6 ± 9.6	0.454
ΔBW1(kg)	5.4 ± 4.1	4.7 ± 4.3	0.456
ΔBW1(%)	9.2 ± 6.4	7.3 ± 7.2	0.262
ΔBW2 (kg)	1.5 ± 4.1	1.1 ± 5.3	0.778
ΔBW2(%)	2.9 ± 8.1	1.9 ± 8.8	0.681
Pre-NLR	4.2 ± 4.2	4.4 ± 4.5	0.878
Post-NLR	9.4 ± 6.1	9.8 ± 7.8	0.867
Rec-NLR	10.1 ± 12.3	6.1 ± 5.0	0.122
ΔNLR1	-5.3 ± 6.9	-5.4 ± 8.2	0.956
ΔNLR2	-1.9 ± .14.1	5.0 ± 7.7	0.028*

SD, standard deviation; BW, body weight; NLR, Neutrophil to lymphocyte ratio; pre-BW, BW before definitive therapy; post-BW, BW after definitive therapy; rec-BW, BW at the detection of recurrence; ΔBW1, pre-BW minus post-BW; delta-BW2, post-BW minus rec-BW; pre-NLR, NLR before definitive therapy; post-NLR, NLR after definitive therapy; rec-NLR, NLR at the detection of recurrence; ΔNLR1, pre-NLR minus post-NLR; ΔNLR2, post-NLR minus rec-NLR; *Significant difference.

**Table 4:** Characteristics of the platinum-refractory and -sensitive recurrence at the detection of recurrence.

	Pt-refractory (n=35), n (%)	Pt-sensitive (n=28), n (%)	P-value
**Locoregional only**			0.717
Asymptomatic	7	4	
Symptomatic	10	8	
**Distant metastasis**			0.004*
Asymptomatic	3	11	
Symptomatic	15	5	

Pt, platinum; *, significant difference.

The univariate and multivariate analyses of OS after the detection of recurrence were presented in **Table 5**. In univariate analysis, time-to-relapse (hazard rate [HR] 0.444, 95% CI: 0.219–0.900, *p* = 0.024), extent of recurrent disease (HR 2.228, 95% CI: 1.094–4.457, *p* = 0.027), salvage treatment (HR 0.238, 95% CI: 0.107–0.531, <0.0001), and salvage surgery (HR 0.430, 95% CI: 0.185–0.995, *p* = 0.049) were associated with a low risk of death. In multivariate analysis, time-to-relapse (HR 0.382, 95% CI: 0.184–0.795, *p* = 0.010) and salvage treatment (HR 0.298, 95% CI: 0.130–0.684, *p* = 0.004) were associated with a low risk of death.

**Table 5:** Univariate and multivariate analysis for OS after the detection of recurrence.

Variate	Univariate analysis			Multivariate analysis		
	HR	95% CI	p-value	HR	95% CI	p-value
Time-to-relapse						
Before 6 months	1	0.219-0.900	0.024*	1	0.184-0.795	0.010*
After 6 months	0.444			0.382		
Extent of recurrent disease						
Locoregional only	1	1.094-4.457	0.027*	1	0.641-4.048	0.310
Distant metastasis	2.228			1.611		
PS						
PS 1or 2	1	0.668-3.942	0.285	–	–	–
PS 3 or 4	1.623			–		
Salvage treatment						
No	1	0.107-0.531	<0.0001*	1	0.130-0.684	0.004*
Yes	0.238			0.298		
Salvage surgery						
No	1	0.185-0.995	0.049*	1	0.187-1.894	0.379
Yes	0.430			0.595		
Salvage radiotherapy						
No	1	0.521-2.246	0.833	–	–	–
Yes	1.082			–		
Systemic therapy						
No	1	0.383-1.749	0.605	–	–	–
Yes	0.818			–		

OS, overall survival; PS, performance status; HR, hazard ratio; CI, confidence interval: *, significant difference.

**Figure 3:**
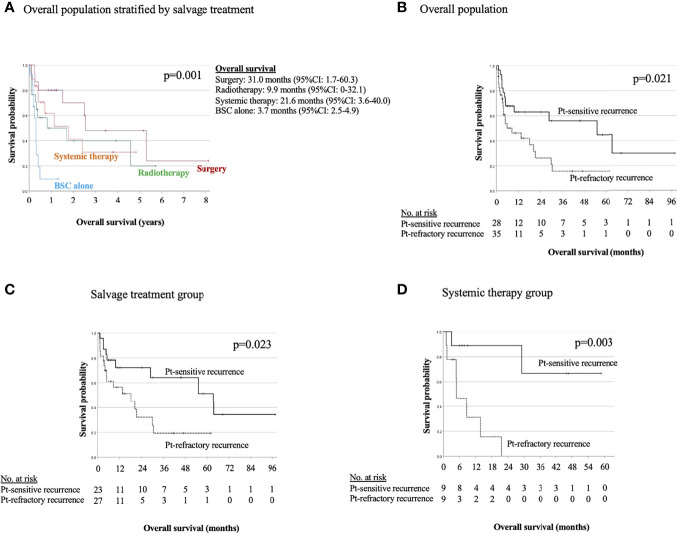
Kaplan−Meier estimates of overall survival in overall population stratified by salvage treatment **(A)** and in overall population **(B)**, salvage treatment group **(C)**, and systemic therapy group **(D)** stratified between the platinum-refractory and platinum-sensitive recurrence.

**Figure 3** shows OS of salvage treatment after the detection of recurrence. In the salvage treatment for recurrence, the longest median OS was 31.0 months (95% CI: 1.7–60.3) for surgery, followed by 21.6 months (95% CI: 3.6–40.0) for systemic therapy, 9.9 months (95% CI: 0–32.1) for RT, and 3.7 months (95%CI: 2.5–4.9) for BSC alone, as shown in **Figure 3** **A**. Salvage therapy and prognosis of patients were analyzed with stratification by the recurrence detection cutoff time of 6 months (**Figures 3** **A–C**). There were significant differences between before and after the cutoff time of 6 months for the overall population (*p* = 0.021), and for salvage treatment group (*p* = 0.023), and systemic therapy group (*p* = 0.003). There were no significant differences between the Pt-refractory and Pt-sensitive recurrence for salvage surgery, RT, and BSC alone in **Supplementary Data 3**.

In the Pt-refractory recurrence, first-line systemic therapy was performed by NIVO in 4 (44.4%) of 9 cases, but second-line systemic therapy was performed in 1 case only (**Supplementary Data 4**). In the Pt-sensitive recurrence, all systemic therapies administered were from 2017 onwards. Of 9 cases, ICI therapy was performed 3 (33.3%) as first-line systemic therapy, 2 (22.2%) as second-line systemic therapy, and 1 (11.1%) as third-line systemic therapy. Second-line systemic therapy was performed in 5 (55.6%) of 9 cases, and third-line systemic therapy was performed in 2 (22.2%) in 9 cases (**Supplementary Data 4**).

## Discussion

This is the first report on pattern of recurrence and efficacy of salvage treatment for recurrence after Pt-containing definitive therapy by CRT or surgery followed by POCRT for L/A SCCHN. Currently, ICI in first-line systemic therapy for uncurable R/M SCCHN is selected by the time-to-relapse from the completion of Pt-containing therapy. Namely, NIVO is selected for Pt-refractory disease, and Pembro is selected for Pt-sensitive disease in R/M SCCHN (2, 3). In Hodgkin’s lymphoma (4–6), small-cell lung carcinoma (7, 8), and ovarian cancer (9–11), it is known that the treatment free survival (TFI) correlates with the response rate to chemotherapy for recurrent disease. The treatment strategies in these malignancies are determined dividing into the refractory or sensitive recurrence stratified the TFI, that is the period between the end of the previous chemotherapy and the start of salvage chemotherapy for relapse, and it differs from the definition of R/M SCCHN. Moreover, the treatment sensitivity and prognosis that are stratified by the time-to-relapse for R/M SCCHN have not been fully investigated. However, recent clinical trials of the ICI therapy have used a time-to-relapse cutoff of 6 months for patient selection (2, 3, 12, 13). Furthermore, the prior treatment of these trials included Pt-containing definitive therapy or no treatment, in addition to chemotherapy treated for uncurable recurrences, and may have included a mix of populations with different biologic characteristics of the target patients. To settle this issue, this study was limited to patients who had relapse after Pt-containing definitive CRT or surgery followed by POCRT.

In this study, 63 patients (42.0%) had locoregional recurrence and/or distant metastasis, and the median time of recurrence was 166 days (95% CI: 117–215). The long-term follow-up data showed that locoregional recurrences developed in 16–25% (14) of patients treated with POCRT for high-risk SCCHN and in 17–52% (15) of patients treated with definitive CRT for unresectable SCCHN. However, these reports did not show the frequency of Pt-refractory or Pt-sensitive recurrence after definitive therapy. The frequency of recurrence of the present study was similar to those reported in previous reports. One of the new findings of this study was Pt-refractory recurrence occurred in 23.3% of cases and Pt-sensitive recurrence occurred in 18.7% of cases after the completion of Pt-containing definitive therapy. The rate of R/M within 1 year after definitive therapy for L/A SCCHN has been reported to be 44% (16); however, 51patients (81.0%) had a relapse within the first year after Pt-containing definitive therapy in this study. This might suggest that recurrence may occur more quickly after Pt-containing definitive therapy, and it may be advisable to follow-up more carefully after treatment. The National Comprehensive Cancer Network guidelines recommend follow-up HN examinations every 1–3 months for the first year after definitive treatment, but the frequency of Pt-refractory or Pt-sensitive recurrence is not described (17). Therefore, this information might be very important for follow-up. Moreover, the occurrence of second primary cancer was 14.7% and 63.6% of them occurred after 2 years from the completion of definitive treatment in this study, which is a factor that requires attention in the follow-up after definitive therapy of L/A SCCHN. Patients with SCCHN have a high risk of developing other cancers simultaneously or subsequently, and the incidence of multiprimary tumors in this population is reported as 27% (18).

This study showed an important finding of the frequency of asymptomatic recurrence according to pattern of recurrence. Asymptomatic recurrence occurred in 37.9% of locoregional recurrence only, 41.2% of distant metastasis, and 63.6% of SPC. Furthermore, asymptomatic distant metastasis after 6 months after definitive therapy occurred significantly more frequently and might require attention. Moreover, it is important to note that 63.6% of SPCs were asymptomatic recurrences and were found after 2 years in 59.0% of cases from the completion of definitive therapy. These suggest the effectiveness of planned imaging examination for follow-up after 6 months from the completion of definitive therapy to detect asymptomatic recurrence; however, the optimal frequency and modality of routine post-treatment imaging in asymptomatic patients is controversial (17). In this study, post-treatment imaging was performed every 3–4 months for the first 2 years, and salvage therapy was promptly performed after recurrence was detected. This timing of imaging examination might be appropriate for detecting recurrence within first year. In addition, more than half of the patients presented with some symptoms, most commonly pain. It is important to note that 71.4% of the cases with Pt-refractory recurrence were symptomatic and that symptomatic distant metastases occurred significantly frequently. Therefore, for patients presenting with symptoms at a clinical visit, the addition of an imaging examination might help in the early detection of recurrence, especially within 6 months after Pt-containing definitive therapy. On the other hand, for asymptomatic patients, planned imaging examination might be especially useful in detecting Pt-sensitive recurrence with distant metastasis and second primary cancer.

In the present study, ΔNLR2 was significantly different between the two cohorts and might be a useful indicator in detecting Pt-refractory recurrence. ΔNLR2 is post-NLR minus rec-NLR and was significantly lower in the Pt-refractory cohort. To the best of our knowledge, no study has examined the use of ΔNLR as a marker of recurrence of SCCHN. In the present study, there was no significant difference in rec-NLR between the two cohorts, but there was a higher NLR in the Pt-refractory cohort compared to the Pt-sensitive cohort. Elevated NLR has been reported to be associated with poor prognosis independent of tumor or disease stage in various cancer types, including HNC (19, 20). NLR is an attractive biomarker for systemic inflammatory responses due to its ease of measurement, low cost, and wide clinical use. This finding might suggest that the Pt-refractory cohort would have a worse prognosis compared to the Pt-sensitive cohort. That is, it might be appropriate to perform frequent measurements of the dynamic change in NLR after Pt-containing definitive therapy, and to perform imaging examination if post-NLR does not decrease. Further studies on the prediction of clinical outcome are needed.

ΔBW1 (BW1%) was reduced by 4.8 kg (7.5%) in the no relapse group, and 5.1 kg (8.4%) in R/M SCCHN group, and 4.7 kg (8.3%) in SPC group, but the difference was not significant. In addition, ΔBW2 (BW2%) was further decreased by 1.4 kg (2.5%) in the Pt-sensitive recurrence and 1.6 kg (3.1%) in the Pt-refractory recurrence, but the difference was not significant. In both cohorts that had relapse, it might be important to note that BW was consistently decreasing throughout the treatment and follow-up periods. It has been reported that weight loss during treatment is associated with poor prognosis (21, 22). Persistent weight loss might be a finding suggestive of recurrence. Further studies on the prediction of recurrence by body weight loss are needed.

In R/M SCCHN, there is a history of clinical trial for systemic chemotherapy in which patients were divided into the Pt-refractory and sensitive groups (2, 3, 12, 13) with an empirical “6 months” cutoff in R/M SCCHN, but the differences in their clinical characteristics and the validity of a cutoff of 6 months have not been fully investigated. In addition, drug sensitivity to platinum agents has long been studied molecularly, with intrinsic resistance and acquired resistance, but biomarkers of platinum resistance in clinical practice are unknown (23, 24). In the present study, a new finding is that multivariate analysis of OS after the detection of recurrence shows time-to-relapse with 6 months cutoff and salvage treatment were associated with a low risk of death, and the median OS in overall population and systemic therapy group between the Pt-refractory and Pt-sensitive recurrence were significantly different. In the salvage treatment group, the Pt-refractory recurrence had a significant poor prognosis compared to the Pt-sensitive recurrence (*p* = 0.023). This indicates that Pt-refractory patients have a poor prognosis even with salvage treatment. Furthermore, there was no significant difference in OS by salvage treatment between the Pt-refractory and Pt-sensitive recurrence, except systemic therapy. We believe that this result support the validity of dividing clinical trials in systemic therapy by time-to-relapse with 6 months cutoff. In systemic therapy of the Pt-sensitive recurrence, ICI was used in 66.7% of the cases, and the median OS was not reached because of the presence of long-term survivors by sequential treatment. All of these patients were selected for systemic therapy in April 2017, when ICIs first became covered by insurance in Japan, and no patients had received systemic therapy prior to that date. This suggests the usefulness of systemic therapy as an option when local treatment is not feasible now that ICI is available. However, Pt-refractory recurrence had a poor prognosis despite the use of NIVO in 44.4% of cases, indicating that patients with Pt-refractory relapse are a treatment-resistant population. Although there were no significant differences, the prognosis for the Pt-sensitive recurrence was good for surgery and radiotherapy compared to the Pt-refractory. The better response to salvage therapy might indicate a better prognosis for Pt-sensitive recurrence compared to the Pt-refractory recurrence. The result suggests that the two populations divided by time-to-relapse differ in their sensitivity to systemic therapy, requiring further exploratory studies.

BSC alone had a worse prognosis compared to salvage treatment, and there was no significant difference between the Pt-refractory and Pt-sensitive recurrence. Indeed, these survival times for BSC alone were roughly equivalent to the 3.82 months reported for natural history of untreated head and neck cancer (25). Multivariate analysis of OS after the detection of recurrence shows salvage treatment was associated with a low risk of death, and it is important for survival in R/M SCCHN that we select the salvage treatment. Surgery is the best salvage treatment in terms of cure and long survival. In addition, if local treatment is not an option, it might be better to treat systemic therapy in terms of long survival in cases with the Pt-sensitive resistance.

Several limitations of this study warrant mention. First, the study was conducted using a retrospective design at a single institution and with a small sample size (*n* = 150). However, to the best of our knowledge, this study is the first to investigate R/M SCCHN divided into Pt-refractory and Pt-sensitive recurrence after Pt-containing definitive therapy. The present results should therefore be considered to provide valuable information for selecting the treatment of R/M SCCHN patients. Second, salvage treatment modalities differ according to historical background, and the short duration of ICI resulted in a different prognosis between the two cohorts. Third, only SCCHN patients with recurrence were included with respect to their course after the completion of Pt-containing definitive therapy in this study. Therefore, although the usefulness of BW and NLR as markers of recurrence of SCCHN is unclear, it is useful to clarify the difference between the Pt-sensitive and Pt-refractory recurrence. Fourth, NLR and BW at 6 months after definitive treatment in the Pt-sensitive cohort were not measured, and differences in ΔNLR between the Pt-refractory and Pt-sensitive cohorts at 6 months after definitive therapy were unknown. However, we believe that the characteristics of Pt-refractory recurrence, in which the NLR that increased with definitive therapy did not decrease, might be clinically useful for the monitoring of recurrence. Dynamic changes in NLR during follow-up require further investigation. Fifth, we used time-to-relapse by the empirical “6 months” cutoff. In systemic therapy, regimens are commonly selected according to the Pt-refractory or Pt-sensitive recurrence as defined by time-to-relapse regimens. Although the biological characteristics of Pt-refractory and Pt-sensitive recurrence have not been clarified, the prognostic difference between the two groups has been revealed, and we consider that this is one of the useful results of this study. However, further investigation of biomarkers of Pt-refractory and Pt-sensitive recurrence is warranted.

## Conclusion

Our analysis revealed the pattern of recurrence after Pt-containing definitive therapy for L/A SCCHN and the frequency of Pt-refractory and Pt-sensitive recurrence. The clinical differences between Pt-refractory and Pt-sensitive recurrence were that ΔNLR2 was lower in the patients with Pt-refractory recurrence, while prognosis was better in those with Pt-sensitive recurrence in salvage treatment and systemic therapy groups. After Pt-containing definitive therapy, it is especially useful in systemic therapy to detect the recurrence by dividing into the Pt-refractory and Pt-sensitive recurrence, which have different prognosis.

## Data Availability Statement

The raw data supporting the conclusions of this article will be made available by the authors, without undue reservation.

## Ethics Statement

Ethical review and approval was not required for the study on human participants in accordance with the local legislation and institutional requirements. The patients/participants provided their written informed consent to participate in this study.

## Author Contributions

TW designed the study and wrote the initial draft of the manuscript. HS contributed to analysis and interpretation of data and assisted in the preparation of the manuscript. All other authors have contributed to data collection and interpretation, and critically reviewed the manuscript. All authors approved the final version of the manuscript, and agree to be accountable for all aspects of the work in ensuring that questions related to the accuracy or integrity of any part of the work are appropriately investigated and resolved.

## Conflict of Interest

The authors declare that the research was conducted in the absence of any commercial or financial relationships that could be construed as a potential conflict of interest.

## Publisher’s Note

All claims expressed in this article are solely those of the authors and do not necessarily represent those of their affiliated organizations, or those of the publisher, the editors and the reviewers. Any product that may be evaluated in this article, or claim that may be made by its manufacturer, is not guaranteed or endorsed by the publisher.
