# Supplementary material for: Pattern of Recurrence After Platinum-Containing Definitive Therapy and Efficacy of Salvage Treatment for Recurrence in Patients with Squamous Cell Carcinoma of the Head and Neck
Source: Front Oncol. 2022 Jul 4;12:876193. doi: 10.3389/fonc.2022.876193 (PMC9289148; doi:10.3389/fonc.2022.876193)
Supplement: Supplementary file 1 [file Table_1.docx]

Supplemental data 1: BW and NLR before definitive therapy, after definitive therapy

|  | No relapse  Average±SD | R/M SCCHN  Average±SD | SPC  Average±SD | P-value |
| --- | --- | --- | --- | --- |
| Pre-BW (kg)  Post-BW (kg)  Rec-BW (kg)  ΔBW1 (kg)  ΔBW1 (%)  ΔBW2 (kg)  ΔBW2 (%) | 58.4±13.3  53.6±11.3  NE  4.8±3.8  7.5±6.3  NE  NE | 57.7±11.2  52.6±9.4  51.4±9.9  5.1±4.2  8.4±6.8  1.3±4.6  2.4±8.3 | 56.3±7.1  51.6±6.9  NE  4.7±2.8  8.3±4.7  NE  NE | 0.695  0.630  0.893  0.826 |
| Pre-NLR  Post-NLR  Rec-NLR  ΔNLR1  ΔNLR2 | 3.7±3.3  8.4±6.3  NE  -4.7±6.7  NE | 4.3±4.3  9.2±6.8  8.0±9.8  -4.9±7.8  1.1±12.1 | 3.2±2.5  5.8±4.2  NE  -2.6±4.2  NE | 0.339  0.849  0.312 |

SD: standard deviation; BW: body weight; NLR: Neutrophil to lymphocyte ratio; pre-BW: BW before definitive therapy; post-BW: BW after definitive therapy; rec-BW: BW at the detection of recurrence; ΔBW1: pre-BW minus post-BW; delta-BW2: post-BW minus rec-BW; pre-NLR: NLR before definitive therapy; post-NLR: NLR after definitive therapy; rec-NLR: NLR at the detection of recurrence; ΔNLR1: pre-NLR minus post-NLR; ΔNLR2: post-NLR minus rec-NLR; NE: not evaluated; *Significant difference.
