# Supplementary material for: Pattern of Recurrence After Platinum-Containing Definitive Therapy and Efficacy of Salvage Treatment for Recurrence in Patients with Squamous Cell Carcinoma of the Head and Neck
Source: Front Oncol. 2022 Jul 4;12:876193. doi: 10.3389/fonc.2022.876193 (PMC9289148; doi:10.3389/fonc.2022.876193)
Supplement: Supplementary file 2 [file Table_2.docx]

**Supplemental data 2.** Univariate analysis of recurrence free survival according to risk factors

|  | HR | 95%CI | p-value |
| --- | --- | --- | --- |
| Sex  Male  Female | 1  0.804 | 0.453-1.426 | 0.455 |
| Disease stage  Ⅲ  　　　　Ⅳ | 1  1.288 | 0.774-2.145 | 0.330 |
| Definitive therapy for primary tumor  Surgery followed by POCRT  CRT | 1  1.120 | 0.699-1.737 | 0.675 |
| Platinum agent used in definitive therapy  Cisplatin  Carboplatin | 1  0.998 | 0.654-1.523 | 0.998 |
| Induction chemotherapy  Yes  No | 1  0.944 | 0.604-1.475 | 0.799 |
| Prophylactic PEG  Yes  No | 1  1.217 | 0.730-2.030 | 0.452 |

HR, hazard ration; CI, confidence interval; PEG, percutaneous endoscopic gastrostomy
