# Supplementary material for: Pattern of Recurrence After Platinum-Containing Definitive Therapy and Efficacy of Salvage Treatment for Recurrence in Patients with Squamous Cell Carcinoma of the Head and Neck
Source: Front Oncol. 2022 Jul 4;12:876193. doi: 10.3389/fonc.2022.876193 (PMC9289148; doi:10.3389/fonc.2022.876193)
Supplement: Supplementary file 3 [file Table_3.docx]

Supplemental data 3. Median OS by salvage treatment in the Pt- refractory and Pt-sensitive cohorts

|  | Pt-refractory recurrence | | Pt-sensitive recurrence | | p-value |
| --- | --- | --- | --- | --- | --- |
|  | n | Median OS (months) (95%CI) | n | Median OS (months) (95%CI) |  |
| Surgery | 9 | 31.0 (17.2-44.7) | 6 | 64.5 (0-153.4) | 0.772 |
| Radiotherapy | 9 | 4.0 (2.6-5.4) | 8 | 55.9 (0-125.2) | 0.160 |
| Systemic therapy | 9 | 5.0 (0.4-9.5) | 9 | Not reached | 0.003* |
| BSC alone | 8 | 3.7 (0.4-7.0) | 5 | 3.8 (2.0-5.5) | 0.430 |

OS: overall survival; BSC: best supportive care; Pt: Platinum, CI: confidence interval; * : significant difference
