# Supplementary material for: Pattern of Recurrence After Platinum-Containing Definitive Therapy and Efficacy of Salvage Treatment for Recurrence in Patients with Squamous Cell Carcinoma of the Head and Neck
Source: Front Oncol. 2022 Jul 4;12:876193. doi: 10.3389/fonc.2022.876193 (PMC9289148; doi:10.3389/fonc.2022.876193)
Supplement: Supplementary file 4 [file Table_4.docx]

Supplemental data 4： Subsequent systemic therapy

|  | First-line, n | Second-line, n | Third-line, n |
| --- | --- | --- | --- |
| Pt-refractory recurrence  n=9 | NIVO 4  Chemo+Cmab 3  Chemo 2 | Chemo 1 | Chemo 1 |
| Pt-sensitive recurrence  n=9 | NIVO 2  Pembro 1  Chemo+Cmab 5  Chemo 1 | NIVO 2  Chemo+Cmab 2  Chemo 1 | NIVO 1  Chemo+Cmab 1 |

NIVO: Nivolumab, Pembro: Pembrolizumab, Chemo: Cytotoxic chemotherapy, Cmab: Cetuximab
